# Supplementary figures and images for: Novel therapeutic strategies for injured endometrium: intrauterine transplantation of menstrual blood‑derived cells from infertile patients
Source: Stem Cell Res Ther. 2023 Oct 15;14:297. doi: 10.1186/s13287-023-03524-z (PMC10577920; doi:10.1186/s13287-023-03524-z)

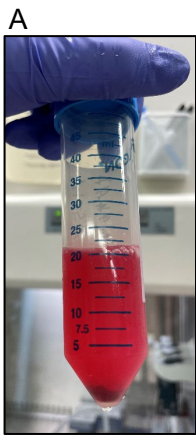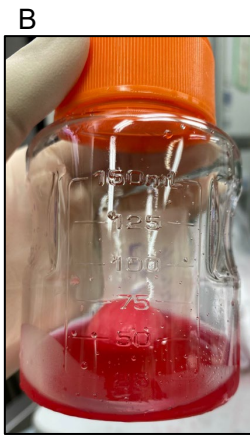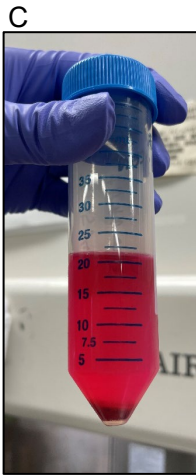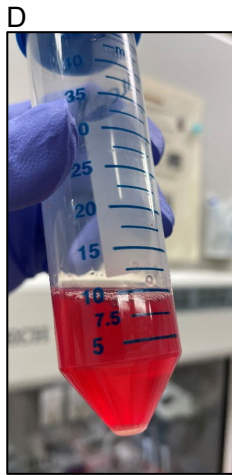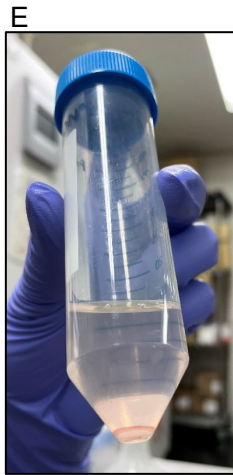

Supplement: Supplementary file 1 — Additional file 1: Figure S1. A collection method of MenSCs from infertile patients. Collection method for menstrual blood samples. Samples were collected into 15-ml tubes with a syringe (A) or a 150-ml bottle with a cotton (B), containing DMEM/2% FBS/1% AA. The sample was centrifuged at 1000 rpm for 5 min (C). After centrifugation, supernatant was removed and 1 ml of DMEM/2% FBS/1% AA was added. Then, 10 ml of RBC lysis buffer was added and gently pipetted several times. After pipetting, the sample was incubated at 4°C for 12 min (D). After incubation, a sample was gently pipetted several times and centrifuged at 1000 rpm for 5 min again. Supernatant was removed, washed with 5 ml of PBS, and centrifuged at 1000 rpm for 5 min. This process was done several times to remove RBC until the supernatant looked transparent (E). [file 13287_2023_3524_MOESM1_ESM.pdf]

A

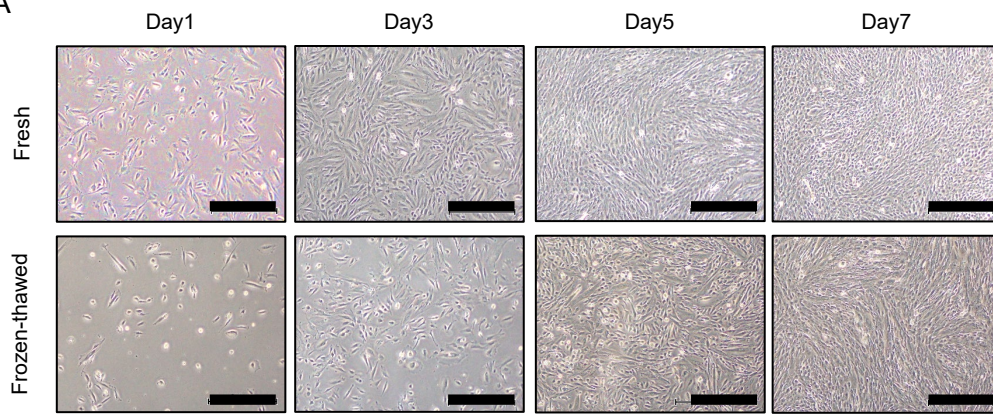

B

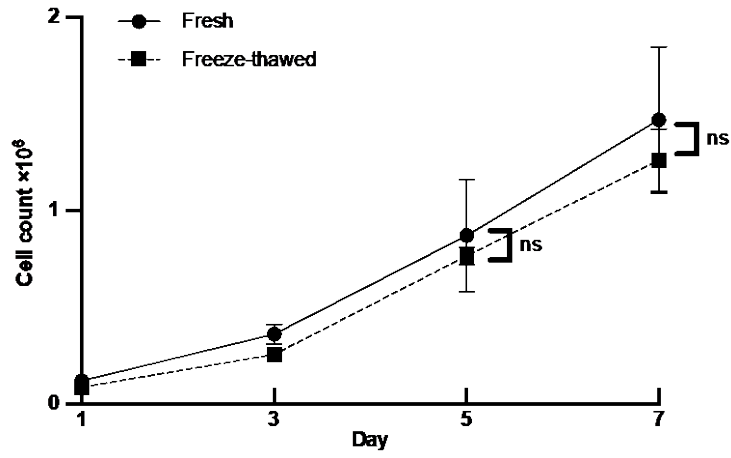

Supplement: Supplementary file 6 — Additional file 6: Figure S2. Proliferative capacity of primary and freeze-thawed MenSCs from infertile patients. (A) Phase-contrast photomicrographs of primary and freeze-thawed MenSCs from infertile patients at different time points. (B) MenSCs (1.0 × 10^5^) at passage 4–7 were plated into 6-well plates with DMEM/10% FBS (n = 3 in each group). Cells were counted at day 1, 3, 5, and 7. Proliferative capacity was not significantly decreased until day 7 even after the freeze-thawed procedure. Statistical significance is shown as *P <0.05 and **P <0.01. 'ns' means 'not significant.' Black bars are 500 μm. [file 13287_2023_3524_MOESM6_ESM.pdf]

A

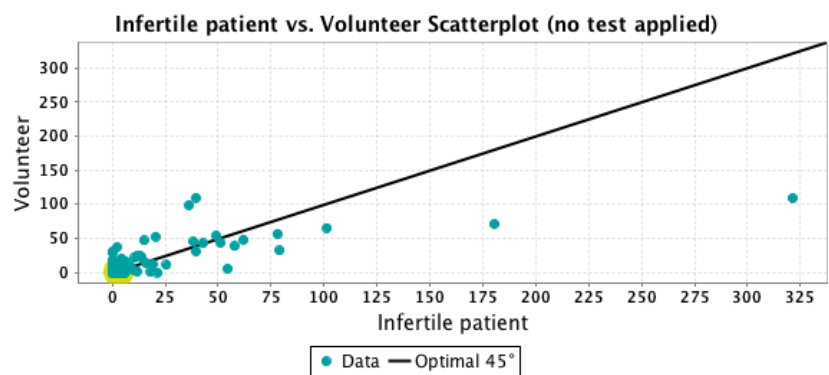

B

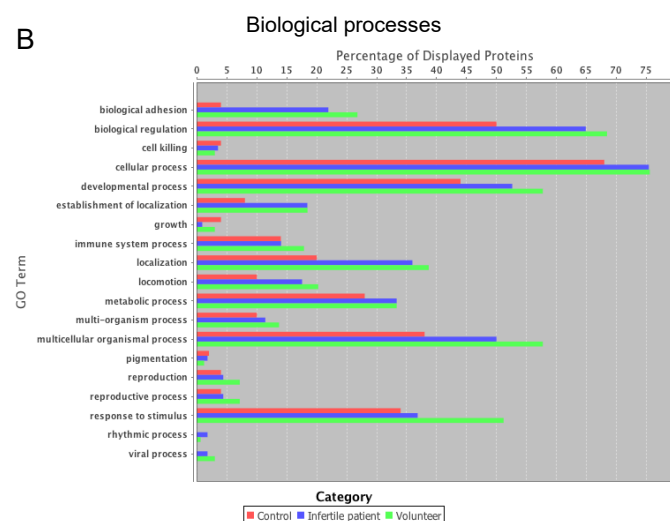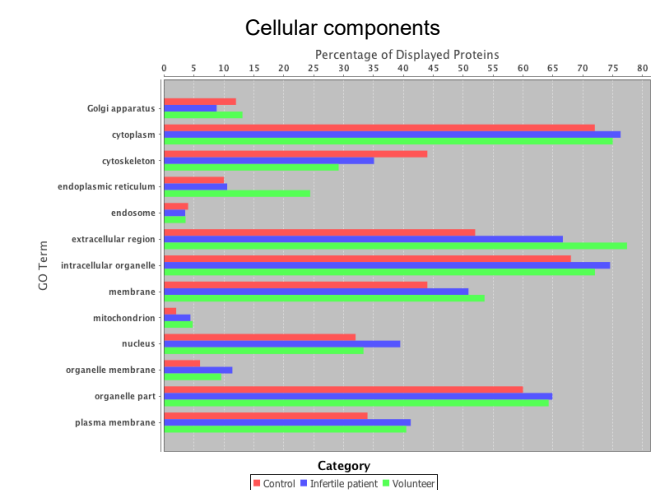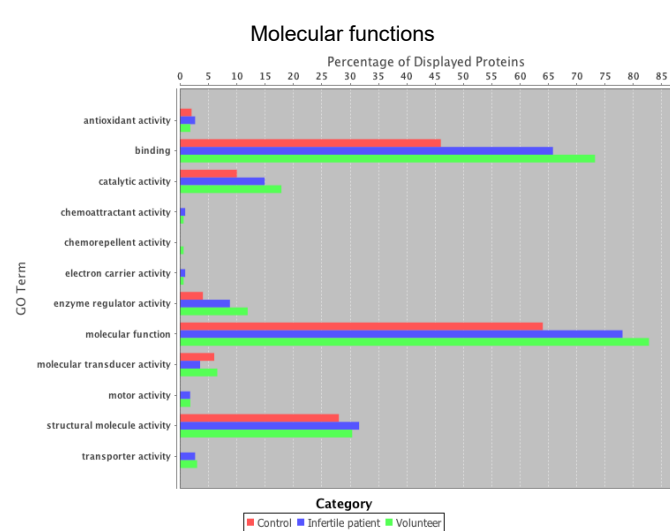

C

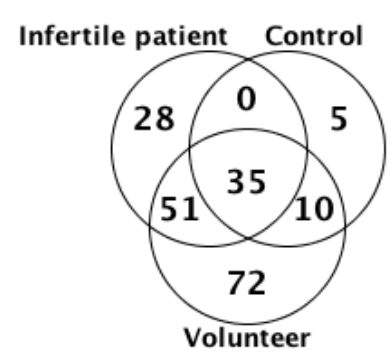

D

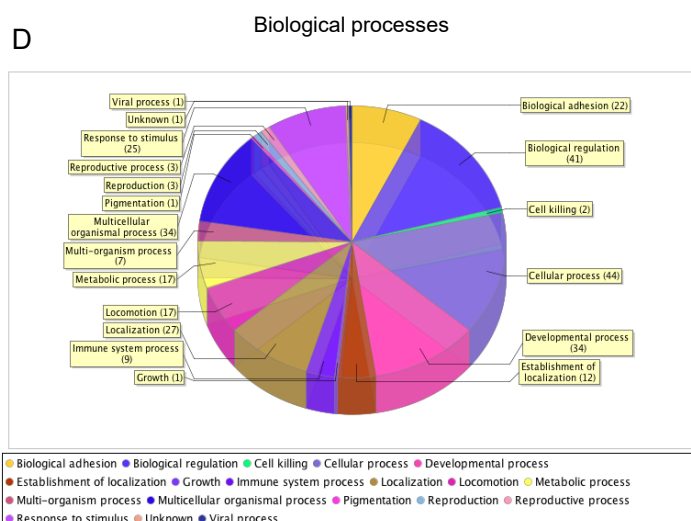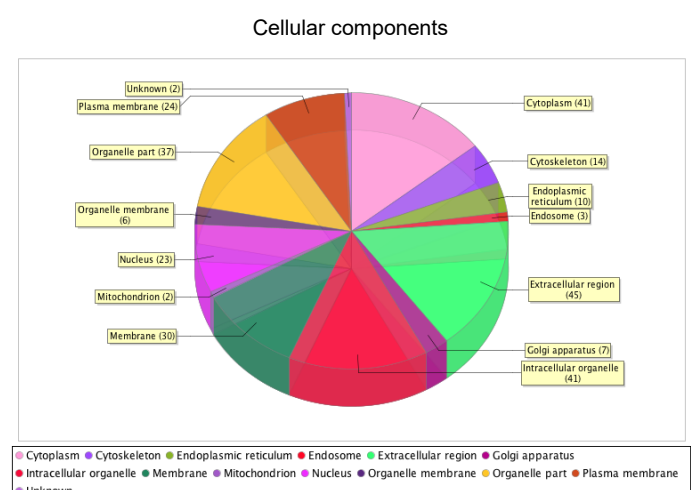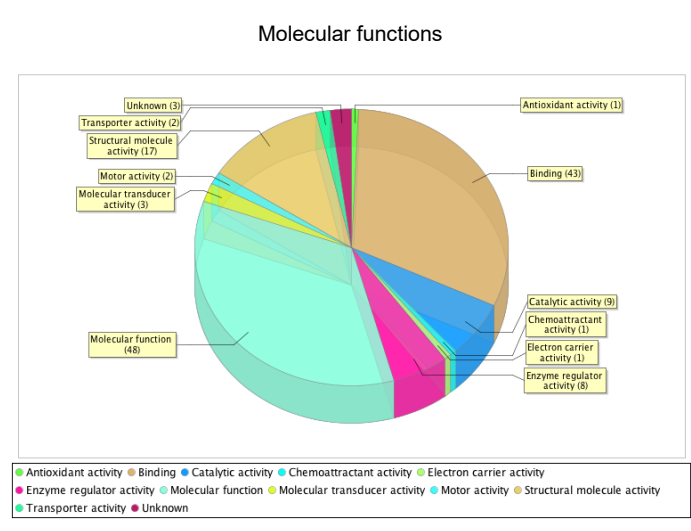

Supplement: Supplementary file 7 — Additional file 7: Figure S3. Proteomics analysis of CM from volunteer-derived and infertile patient-derived MenSCs. (A) Quantitative scatterplot indicated that proteins contained in CM from volunteer-derived and infertile patient-derived MenSCs were similar to each other. (B) Number of 'unique proteins' was similar in the volunteer-derived and infertile patient-derived CM in the categories of 'biological process,' 'cellular component,' and 'molecular function.' (C) Venn diagram showing that total 51 proteins were common between volunteer-derived and infertile patient-derived CM. (D) The 51 proteins were primarily categorized into subgroups of 'biological process' such as 'cellular process,' 'biological regulation,' 'developmental process,' 'multicellular organismal process,' 'response to stimulus,' 'localization,' and 'biological adhesion.' [file 13287_2023_3524_MOESM7_ESM.pdf]

A

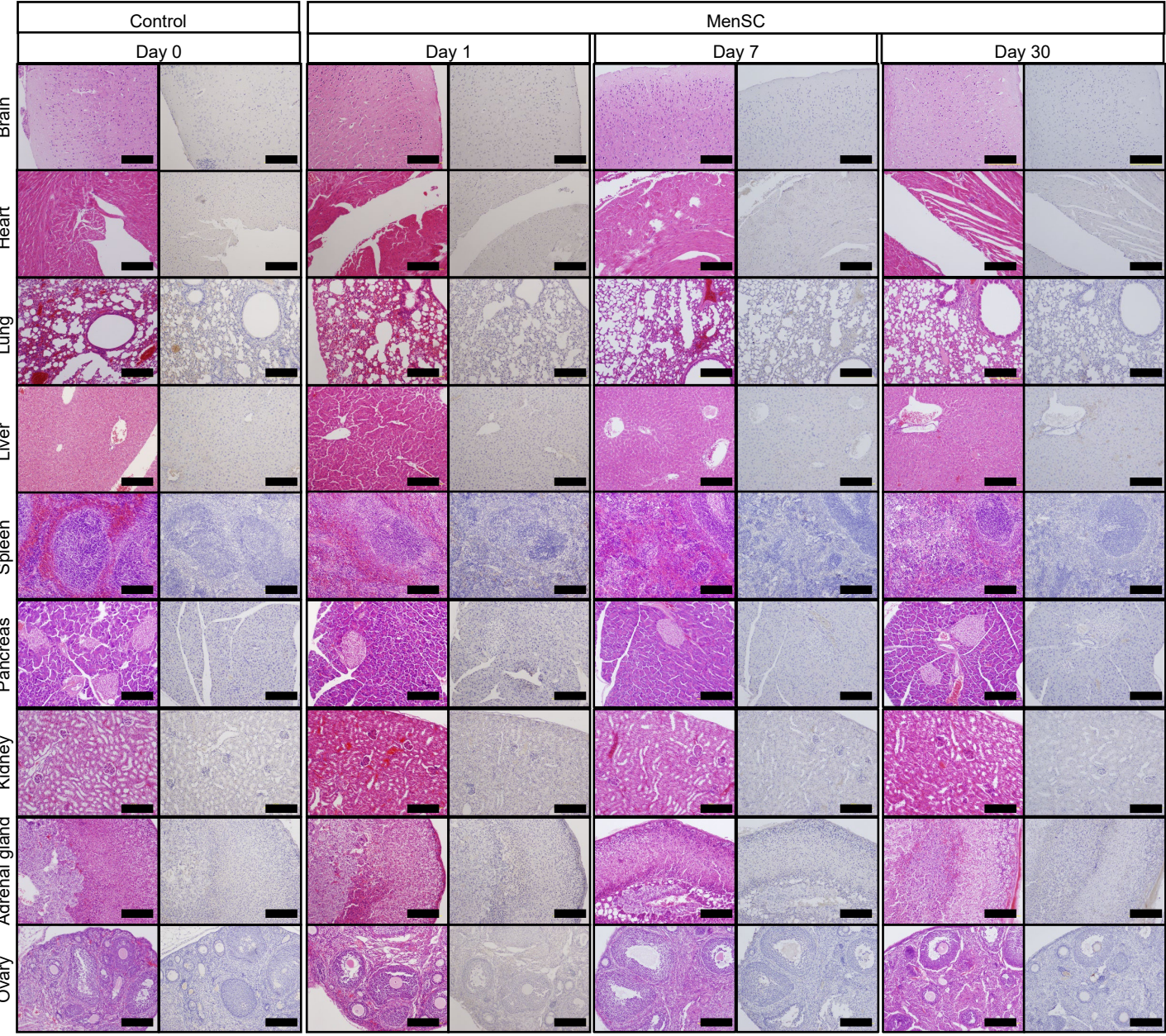

Supplement: Supplementary file 10 — Additional file 10: Figure S4. Systemic biodistribution of intrauterine transplanted MenSCs in major organs. Histopathological findings of major organs at day 1, 7, and 30 after intrauterine transplantation of MenSCs (n = 3 in each group). There were no human vimentin-positive cells and tumor formation in brain, heart, lungs, liver, spleen, pancreas, kidneys, adrenal glands, and ovaries. Black bars are 500 μm. [file 13287_2023_3524_MOESM10_ESM.pdf]

A

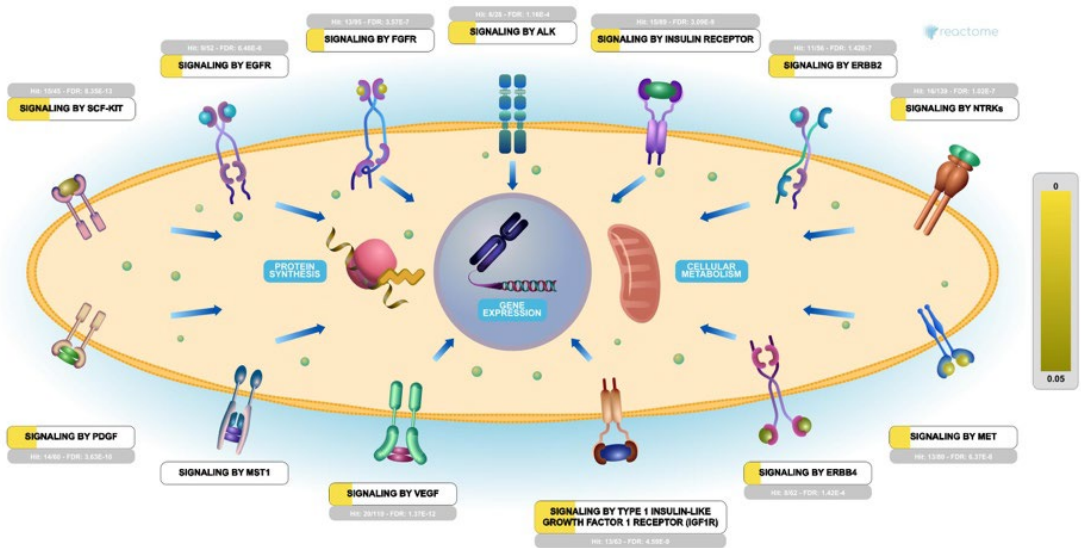

Supplement: Supplementary file 13 — Additional file 13: Figure S5. Pathway analysis using Reactome Pathway Database. Pathways were analyzed for the shared proteins in the conditioned media between infertile patient-derived and volunteer-derived MenSCs using Reactome Pathway Database. The conditioned media contained proteins associated with pathways for cell growth and neovascularization. P < 0.05 was defined as statistically significance. [file 13287_2023_3524_MOESM13_ESM.pdf]
