# Supplementary material for: Novel therapeutic strategies for injured endometrium: intrauterine transplantation of menstrual blood‑derived cells from infertile patients
Source: Stem Cell Res Ther. 2023 Oct 15;14:297. doi: 10.1186/s13287-023-03524-z (PMC10577920; doi:10.1186/s13287-023-03524-z)
Supplement: Supplementary file 2 — Additional file 2: Table S1. List of antibodies for immunohistochemistry and flow-cytometry [file 13287_2023_3524_MOESM2_ESM.pdf]

Supplemental Table 1. List of antibodies for immunohistochemistry and flow-cytometry

Name	Clone	Species reactivity	Host	Company	Dilution
Immunohistochemistry					
Primary antibodies					
Anti-CD34 antibody	Ab81289	Mouse, rat, human	Rabbit, Monoclonal IgG	Abcam	1/2500
Anti-Ki-67 antibody	Ab15581	Mouse, human	Rabbit, Polyclonal IgG	Abcam	1/300
Anti-human Vimentin antibody	M7020	human	Mouse, Monoclonal IgG	Dako	1/100
Secondary antibodies					
-Cellstain®- DAPI solution	D523	None		DOJINDO	1/1000
Rabbit anti-goat IgG Secondary antibody, Alexa Fluor 488	A11078	None		Invitrogen	1/500
Goat anti-mouse IgG1 Secondary antibody, Alexa Fluor 546	A21123	None		Invitrogen	1/500
Flow-cytometry					
Anti-CD73 Antibody, anti-human, REAfinity™	AD2	human	human cell line, monoclonal IgG1	Miltenyi Biotec Inc	1/50
Anti-CD90 Antibody, anti-human, REAfinity™	DG3	human	human cell line, monoclonal IgG1	Miltenyi Biotec Inc	1/50
Anti-CD105 Antibody, anti-human, REAfinity™	43A4E1	human	human cell line, monoclonal IgG1	Miltenyi Biotec Inc	1/50
Anti-CD14 Antibody, anti-human, REAfinity™	REA599	human	human cell line, monoclonal IgG1	Miltenyi Biotec Inc	1/50
Anti-CD19 Antibody, anti-human, REAfinity™	REA675	human	human cell line, monoclonal IgG1	Miltenyi Biotec Inc	1/50
Anti-CD34 Antibody, anti-human, REAfinity™	REA1164	human	human cell line, monoclonal IgG1	Miltenyi Biotec Inc	1/50
Anti-CD45 Antibody, anti-human, REAfinity™	REA747	human	human cell line, monoclonal IgG1	Miltenyi Biotec Inc	1/50
Anti-HLA-DR Antibody, anti-human, REAfinity™	REA805	human	human cell line, monoclonal IgG1	Miltenyi Biotec Inc	1/50
Isotype Control Antibody mouse IgG1, PE	IS5-21F5	human	mouse, monoclonal IgG1	Miltenyi Biotec Inc	1/50
REA Control Antibody(S), human IgG1, APC, REAfinity	REA293	human	human cell line, monoclonal IgG1	Miltenyi Biotec Inc	1/50

DAPI, 4',6-Diamidino-2-Phenylindole, dihydrochloride.
