# Supplementary material for: Novel therapeutic strategies for injured endometrium: intrauterine transplantation of menstrual blood‑derived cells from infertile patients
Source: Stem Cell Res Ther. 2023 Oct 15;14:297. doi: 10.1186/s13287-023-03524-z (PMC10577920; doi:10.1186/s13287-023-03524-z)
Supplement: Supplementary file 3 — Additional file 3: Table S2. List of primer sequences for quantitative reverse transcription polymerase chain reaction [file 13287_2023_3524_MOESM3_ESM.pdf]

Supplemental Table 2. List of primer sequences for quantitative reverse transcription polymerase chain reaction

Gene	Primer sequence	5' to 3'
VEGFA	Forward	5' TCAGTTCGAGGAAAGGGAAA 3'
	Reverse	5' GCGAGTCTGTGTTTTTGCAG 3'
FGF-1	Forward	5' GGACACCGAAGGGCTTTTAT 3'
	Reverse	5' ACAGCTCCCGTTCTTCTTGA 3'
FGF-2	Forward	5' CCTTGCTATGAAGGAAGATGG 3'
	Reverse	5' CCGTTTTGGATCCGAGTTTA 3'
EGF	Forward	5' CGTGTGCATGCATATTGAATC 3'
	Reverse	5' TTCCCACCATCGTAGGTCTC 3'
GAPDH	Forward	5' TGTTGCCATCAATGACCCCTT 3'
	Reverse	5' CTCCACGACGTACTCAGCG 3'

VEGFA, vascular endothelial growth factor A; FGF, fibroblast growth factor; EGF, epidermal growth factor; GAPDH, glyceraldehyde 3-phosphate dehydrogenase.
