# Supplementary material for: Novel therapeutic strategies for injured endometrium: intrauterine transplantation of menstrual blood‑derived cells from infertile patients
Source: Stem Cell Res Ther. 2023 Oct 15;14:297. doi: 10.1186/s13287-023-03524-z (PMC10577920; doi:10.1186/s13287-023-03524-z)
Supplement: Supplementary file 4 — Additional file 4: Table S3. Patient characteristics of samples with unsuccessful primary culture of menstrual-blood derived cells [file 13287_2023_3524_MOESM4_ESM.pdf]

Supplemental Table 3. Patient characteristics of samples with unsuccessful primary culture of menstrual blood-derived cells

Sample #	Age range, y	Gravida	Parity	BMI, kg/m^2	Cause for infertility	Duration of infertility, y	Treatment method	Menstrual day	Collection method
5	45-49	1	1	23.3	Ovarian insufficiency	0.5	TI	D3	Cotton
7	35-39	2	1	21.1	PCOS	1.1	TI	D2	Cotton
11	40-44	4	1	19.6	Endometriosis	0.8	ART	D5	Cotton
12	35-39	0	0	19.7	Unexplained	0.4	TI	D3	Cotton
15	30-34	0	0	20.1	PCOS	0.6	IUI	D4	Syringe
16	35-39	0	0	18.1	PCOS	0.7	TI	D3	Cotton

y, year; BMI, body mass index; PCOS, polycystic ovary syndrome; TI, timed intercourse; ART, assisted reproductive technology; IUI, intrauterine insemination; D, day.
