# Supplementary material for: Novel therapeutic strategies for injured endometrium: intrauterine transplantation of menstrual blood‑derived cells from infertile patients
Source: Stem Cell Res Ther. 2023 Oct 15;14:297. doi: 10.1186/s13287-023-03524-z (PMC10577920; doi:10.1186/s13287-023-03524-z)
Supplement: Supplementary file 5 — Additional file 5: Table S4. Flow cytometric analysis for mesenchymal stem cell markers [file 13287_2023_3524_MOESM5_ESM.pdf]

Supplemental Table 4. Flow cytometric analysis for mesenchymal stem cell markers

Antigen	Volunteers (%, mean ± SD)	Infertile patients (%, mean ± SD)	*P* -value
CD73	96.07 ± 4.31	98.80 ± 0.36	0.33
CD90	85.57 ± 6.93	75.83 ± 11.93	0.48
CD105	94.63 ± 4.14	96.63 ± 3.50	0.66
CD14	0.70 ± 0.63	1.03 ± 0.25	0.49
CD19	0.73 ± 0.61	0.98 ± 0.21	0.57
CD34	0.70 ± 0.64	1.26 ± 0.54	0.44
CD45	0.83 ± 0.42	1.45 ± 0.28	0.16
HLA-DR	0.65 ± 0.54	0.89 ± 0.04	0.48

These measurements were conducted entirely independent of all other variables (n=3). Student's t-test was conducted for calculating statistical difference. $P < 0.05$ was defined as statistically significance.
