# Supplementary material for: Novel therapeutic strategies for injured endometrium: intrauterine transplantation of menstrual blood‑derived cells from infertile patients
Source: Stem Cell Res Ther. 2023 Oct 15;14:297. doi: 10.1186/s13287-023-03524-z (PMC10577920; doi:10.1186/s13287-023-03524-z)
Supplement: Supplementary file 11 — Additional file 11: Table S6. Body and organ weights of the mice in each group [file 13287_2023_3524_MOESM11_ESM.pdf]

Supplemental Table 6. Body and organ weights of the mice in each group

Variables, g, n=4	Sham	Injured	MenSC	*P*- value
Body weight	27.70 ± 2.37	28.11 ± 2.03	27.29 ± 0.93	0.82
Body weight change	-0.18 ± 0.88	0.23 ± 0.92	0.52 ± 0.52	0.48
Brain	0.489 ± 0.045	0.523 ± 0.029	0.479 ± 0.039	0.28
Heart	0.149 ± 0.016	0.154 ± 0.004	0.144 ± 0.014	0.51
Lung	0.204 ± 0.044	0.205 ± 0.038	0.194 ± 0.003	0.99
Liver	1.89 ± 0.11	1.92 ± 0.19	1.98 ± 0.17	0.74
Spleen	0.120 ± 0.024	0.122 ± 0.023	0.116 ± 0.028	0.95
Pancreas	0.242 ± 0.061	0.221 ± 0.042	0.235 ± 0.064	0.86
Kidney	0.474 ± 0.041	0.488 ± 0.039	0.486 ± 0.040	0.86
Adrenal gland	0.014 ± 0.003	0.009 ± 0.002	0.010 ± 0.003	0.09
Ovary	0.019 ± 0.005	0.028 ± 0.004	0.023 ± 0.007	0.13

Data are presented as mean ± SD. One-way ANOVA test was conducted for calculating statistical difference. P <0.05 was defined as statistically significance.
