# Supplementary material for: Novel therapeutic strategies for injured endometrium: intrauterine transplantation of menstrual blood‑derived cells from infertile patients
Source: Stem Cell Res Ther. 2023 Oct 15;14:297. doi: 10.1186/s13287-023-03524-z (PMC10577920; doi:10.1186/s13287-023-03524-z)
Supplement: Supplementary file 12 — Additional file 12: Table S7. Blood sample test of the mice in each group [file 13287_2023_3524_MOESM12_ESM.pdf]

Supplemental Table 7. Blood sample test of the mice in each group

Variables, n=4	Sham	Injured	MenSC	*P*-value
WBC (×10^2/μL)	47.50 ± 14.53	45.75 ± 11.44	27.75 ± 9.39	0.07
RBC (×10^4/μL)	825.25 ± 66.83	801.75 ± 82.27	767.50 ± 14.98	0.44
Hb (g/dL)	13.32 ± 1.07	13.00 ± 1.43	12.43 ± 0.40	0.50
HCT (%)	40.45 ± 3.78	39.18 ± 3.58	37.13 ± 1.41	0.35
MCV (fL)	48.9 ± 0.96	48.93 ± 1.74	48.38 ± 1.06	0.80
MCH (pg)	16.15 ± 0.12	16.20 ± 0.30	16.18 ± 0.16	0.98
MCHC (g/dL)	33.00 ± 0.65	33.15 ± 0.57	33.45 ± 0.24	0.48
PLT (×10^4/μL)	111.85 ± 20.81	108.75 ± 6.40	121.25 ± 4.03	0.92
TP (g/dL)	4.98 ± 0.33	4.98 ± 0.26	4.73 ± 0.30	0.42
A/G ratio	1.68 ± 0.21	1.60 ± 0.22	1.83 ± 0.05	0.23
Alb (g/dL)	3.10 ± 0.14	3.03 ± 0.10	3.05 ± 0.17	0.75
BUN (mg/dL)	26.53 ± 4.23	22.80 ± 1.24	23.48 ± 2.09	0.19
Cre (mg/dL)	0.11 ± 0.02	0.09 ± 0.01	0.10 ± 0.01	0.45
Na (mEq/L)	148.00 ± 4.08	149.75 ± 2.36	148.25 ± 4.35	0.77
Cl (mEq/L)	108.75 ± 2.63	109.75 ± 1.89	108.00 ± 1.83	0.53
ALT (IU/L)	21.00 ± 4.97	21.00 ± 5.03	27.75 ± 5.38	0.19
ALP (IU/L)	248.25 ± 19.24	312.50 ± 75.54	254.25 ± 93.27	0.39

Data are presented as mean ± SD. One-way ANOVA test was conducted for calculating statistical difference. P <0.05 was defined as statistically significance.
